# Diagnostic Pathology – after two years

**DOI:** 10.1186/1746-1596-3-1

**Published:** 2008-01-04

**Authors:** Klaus Kayser

**Affiliations:** 1 Institute of Pathology, Charite, Humboldt University, Berlin, Germany

## Abstract

With the second anniversary of *Diagnostic Pathology* approaching, this Editorial looks at the journal's achievements so far and outlines the prospects and challenges.

## Editorial

Dear Friends, Colleagues, and Authors,

Please allow me to express my deep gratitude for your interest in and becoming part of our still fairly young enterprise *Diagnostic Pathology*. The journal's success so far encourages me to ask for your continued support for implementing a high-quality scientific journal covering the whole range of diagnostic pathology. Our aim is to provide clinical colleagues and researchers working in medical diagnostics and therapy with a high-quality, innovative, and easy-to-handle instrument for the distribution of new insights into tissue-based medical science. Topics include all aspects of medical information that can be acquired from human tissue by relevant technologies such as light or electron microscopy, blood analysis, polymerase chain reaction methodology, gene analysis, and data mining, and we also consider descriptions of individual manifestations of disease.

Our open access, internet-based journal can be read by anyone at any place of the globe without payment or other access barriers, the only condition being internet access. From our point of view this constellation especially supports colleagues and researchers working at places or in countries that might experience financial difficulties in accessing traditional subscription-based journals. *Diagnostic Pathology*, on the other hand, is made sustainable by author-side charges which can be covered by research grants, financial support from the rapidly growing number of institutions welcoming open access, and institutions' arrangements with BioMed Central as the journal's publisher. BioMed Central also operates a waiver policy so as to ensure that publication charges do not impose new barriers after access barriers have come down. We fully acknowledge the efforts of authors, funders, and everyone else involved; we are very grateful as they really give us the opportunity to promote our journal *Diagnostic Pathology *and the unique opportunities it provides for the rapid and efficient dissemination of knowledge in our field.

*Diagnostic Pathology *is coming up to its second anniversary, and we can retrospectively analyze the initial two years. On average, we had about 70 – 75 submitted articles per year. At present we can accept about two thirds of them, with one third being rejected. All in all, an average of four articles per month is published. Our Editorial Board of well-known experts works hard to meet the aim of assuring the scientific quality of submitted articles and to provide authors with a first response as soon as possible, often within 14 days after submission. The board consists of active young experts working alongside senior scientists of international standing. The authors can expect that their article will be published within two months or even less, counted from first submission. Where it takes longer, this is usually due to authors needing more time to revise their papers based on the reviewers' recommendations. We would like to take the opportunity to thank all reviewers for their thorough and entirely neutral evaluations of submitted articles.

Especially over the last year, the publisher has improved the tools for the submission and handling of articles, in close agreement with our suggestions. We are still engaged in further developing the technical features and to implement new ideas so as to benefit authors, editors, reviewers, and readers. Some ideas have been suggested in our previous Editorial. These included especially a close contact with our readers, whom we encourage to participate in discussions of and comments on published articles. We are convinced that this interactive publication model will be of additional benefit for both authors and readers. The first step would be an implementation of a discussion forum associated with already published articles. The next step under discussion is the implementation of virtual slides in connection with a specific server. Basically, this idea will connect science with education and will open a new chapter in the distribution of medical scientific data.

Several readers (and authors) have asked us about indexing coverage of our journal. At present, we are in the evaluation process for impact factor tracking by Thomson Scientific. Also, we are optimistic about meeting the conditions and to be approved for inclusion in Medline in the near future. However, independent of indexing and tracking by external services we can provide our authors and readers with access data (number of times articles are viewed). We are proud about the fact that more than seven articles display more than 300 clicks per months on average. Several articles have been accessed more than 4000 times since 2006 – and this is only counting accesses to BioMed Central's server, a figure that would easily double if downloads from PubMed Central and other freely accessible full-text repositories were added. We can therefore assure our authors that their articles will be noticed by vast numbers of researchers, practising clinicians and not least lay readers (including patients), and that our journal is already established and recognized by colleagues interested in our work in the field of diagnostic pathology.

The journal considers a broad range of article types, such as research, reviews, case reports, methodology articles, debates, or hypotheses. A high percentage of the articles published is made up of case reports, most of them with very rare diseases or disease constellations. We believe that case reports are still of scientific and educational value, even – or especially – in the time of electronic communication and evidence-based learning. Usually, at least half of the most-viewedarticles are case reports, vindicating the value of case reports for the community. Keeping the balance right between scientific quality, medical interest, and working conditions of authors who want to demonstrate their experience and expertise is a major factor in building a successful scientific journal such as *Diagnostic Pathology*. This duty has been successfully supported by all members of the Editorial Board, and my special gratitude goes to Dr. Torsten Goldmann and Dr. Gian Kayser. In addition, *Diagnostic Pathology *has been excellently assisted by the editorial staff at Biomed Central, especially by Stefan Busch and his colleagues whom I thank very much for all their efforts.

Dear authors and readers, let us work together to further improve *Diagnostic Pathology *with the aim to create an increasingly attractive, innovative and cutting-edge online publication for the benefit of the community.

I wish all our readers, authors, interested scientists, and members of our Editorial Board a Happy, Healthy and Prosperous New Year 2008.

Berlin, December 2007

Klaus Kayser M.D., Ph.D.

Editor-in-Chief.

